# Supplementary material for: Nitrogen Acquisition and Transport in the Ectomycorrhizal Symbiosis—Insights from the Interaction between an Oak Tree and Pisolithus tinctorius
Source: Plants (Basel). 2022 Dec 20;12(1):10. doi: 10.3390/plants12010010 (PMC9823632; doi:10.3390/plants12010010)
Supplement: Supplementary file 1 [file plants-12-00010-s001.zip › Figure S1.pptx]

## Slide 1
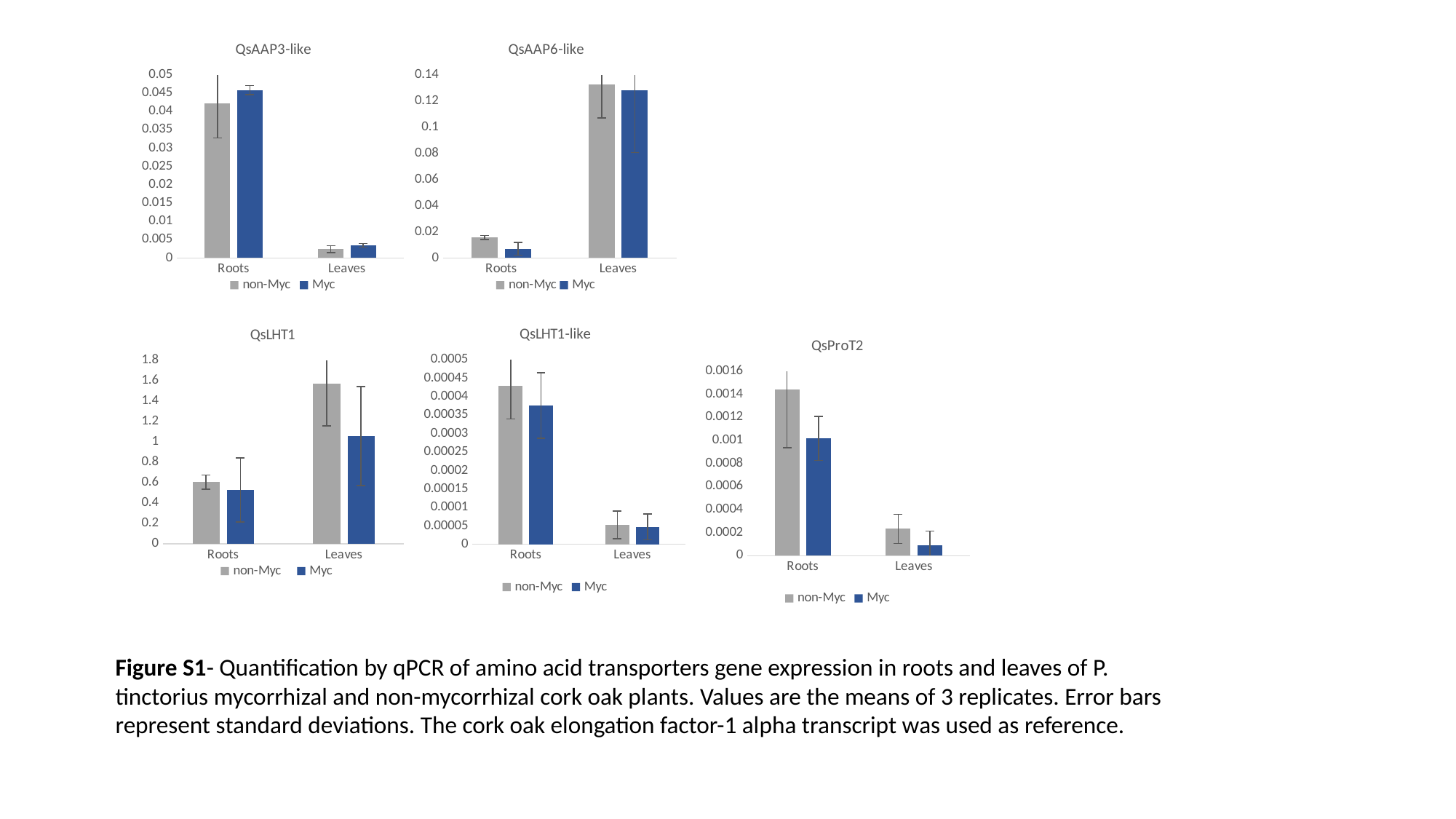

### Chart: QsAAP3-like
| Category | non-Myc | Myc |
|---|---|---|
| Roots | 0.04227104401382816 | 0.04583674195172626 |
| Leaves | 0.0024186354830752323 | 0.0034677361853960712 |
### Chart: QsAAP6-like
| Category | non-Myc | Myc |
|---|---|---|
| Roots | 0.015569496434201269 | 0.00695929365267176 |
| Leaves | 0.13242581400348583 | 0.12848110127406678 |
### Chart: QsLHT1-like
| Category | non-Myc | Myc |
|---|---|---|
| Roots | 0.0004295001030252288 | 0.00037592252626491955 |
| Leaves | 5.257798310548986e-05 | 4.721868184333444e-05 |
### Chart: QsLHT1
| Category | non-Myc | Myc |
|---|---|---|
| Roots | 0.6045833069743894 | 0.5280715797356299 |
| Leaves | 1.570768520020814 | 1.0558887235115366 |
### Chart: QsProT2
| Category | non-Myc | Myc |
|---|---|---|
| Roots | 0.0014418343661315592 | 0.001016046769382813 |
| Leaves | 0.00023186891675882593 | 8.823331375111814e-05 |Figure S1- Quantification by qPCR of amino acid transporters gene expression in roots and leaves of P. tinctorius mycorrhizal and non-mycorrhizal cork oak plants. Values are the means of 3 replicates. Error bars represent standard deviations. The cork oak elongation factor-1 alpha transcript was used as reference.
