# Supplementary material for: Nitrogen Acquisition and Transport in the Ectomycorrhizal Symbiosis—Insights from the Interaction between an Oak Tree and Pisolithus tinctorius
Source: Plants (Basel). 2022 Dec 20;12(1):10. doi: 10.3390/plants12010010 (PMC9823632; doi:10.3390/plants12010010)
Supplement: Supplementary file 1 [file plants-12-00010-s001.zip › Figure S2.pptx]

## Slide 1
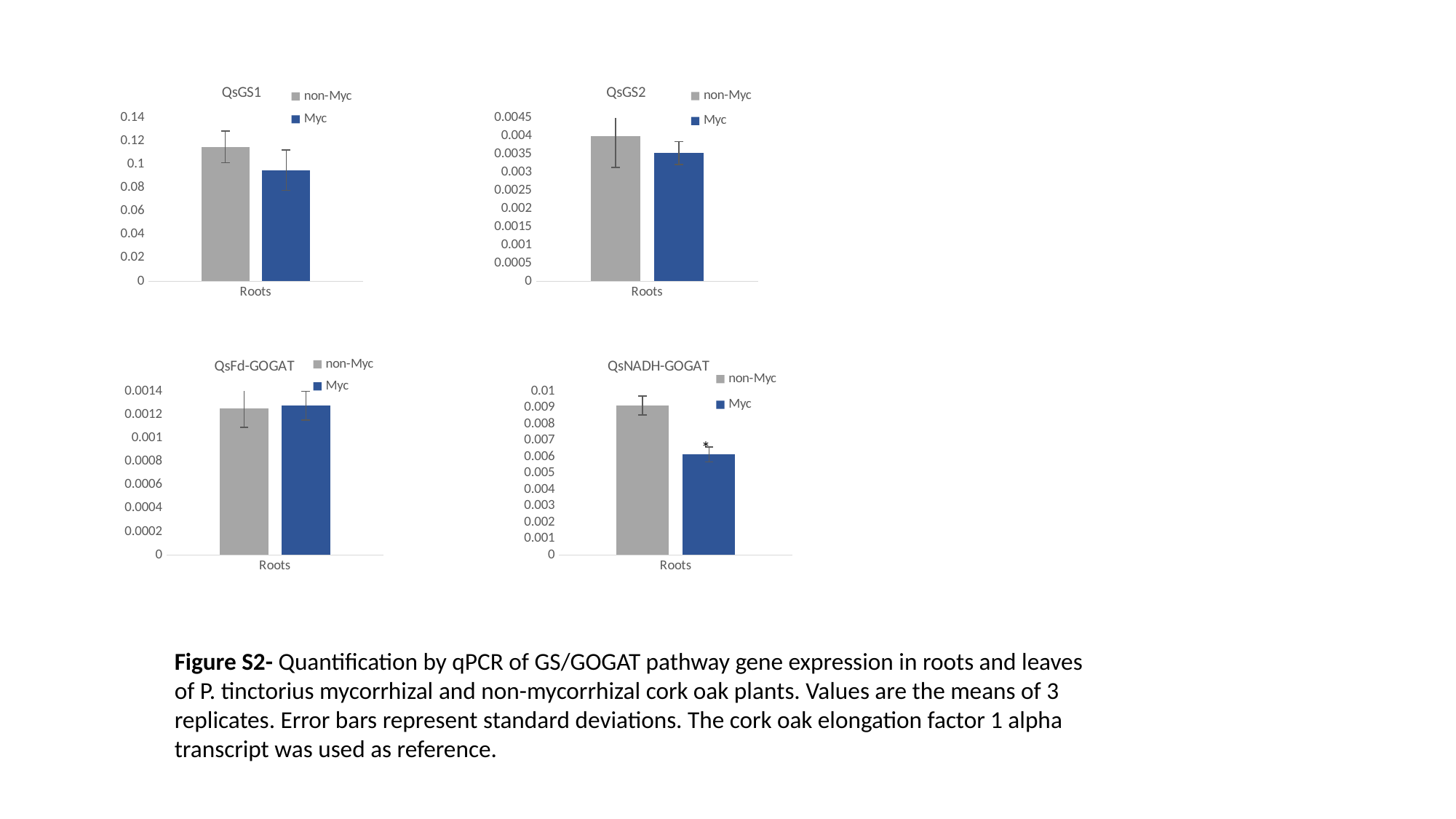

### Chart: QsGS1
| Category | non-Myc | Myc |
|---|---|---|
| Roots | 0.11495148702667368 | 0.09505543982993496 |
### Chart: QsGS2
| Category | non-Myc | Myc |
|---|---|---|
| Roots | 0.003996602769465248 | 0.0035275054186945363 |
### Chart: QsFd-GOGAT
| Category | non-Myc | Myc |
|---|---|---|
| Roots | 0.0012531633081527861 | 0.001276805540387982 |
### Chart: QsNADH-GOGAT
| Category | non-Myc | Myc |
|---|---|---|
| Roots | 0.009125417724875502 | 0.006134204060491998 |*
Figure S2- Quantification by qPCR of GS/GOGAT pathway gene expression in roots and leaves of P. tinctorius mycorrhizal and non-mycorrhizal cork oak plants. Values are the means of 3 replicates. Error bars represent standard deviations. The cork oak elongation factor 1 alpha transcript was used as reference.
