# Supplementary material for: Nitrogen Acquisition and Transport in the Ectomycorrhizal Symbiosis—Insights from the Interaction between an Oak Tree and Pisolithus tinctorius
Source: Plants (Basel). 2022 Dec 20;12(1):10. doi: 10.3390/plants12010010 (PMC9823632; doi:10.3390/plants12010010)
Supplement: Supplementary file 1 [file plants-12-00010-s001.zip › Figure S4_Revised.pptx]

## Slide 1
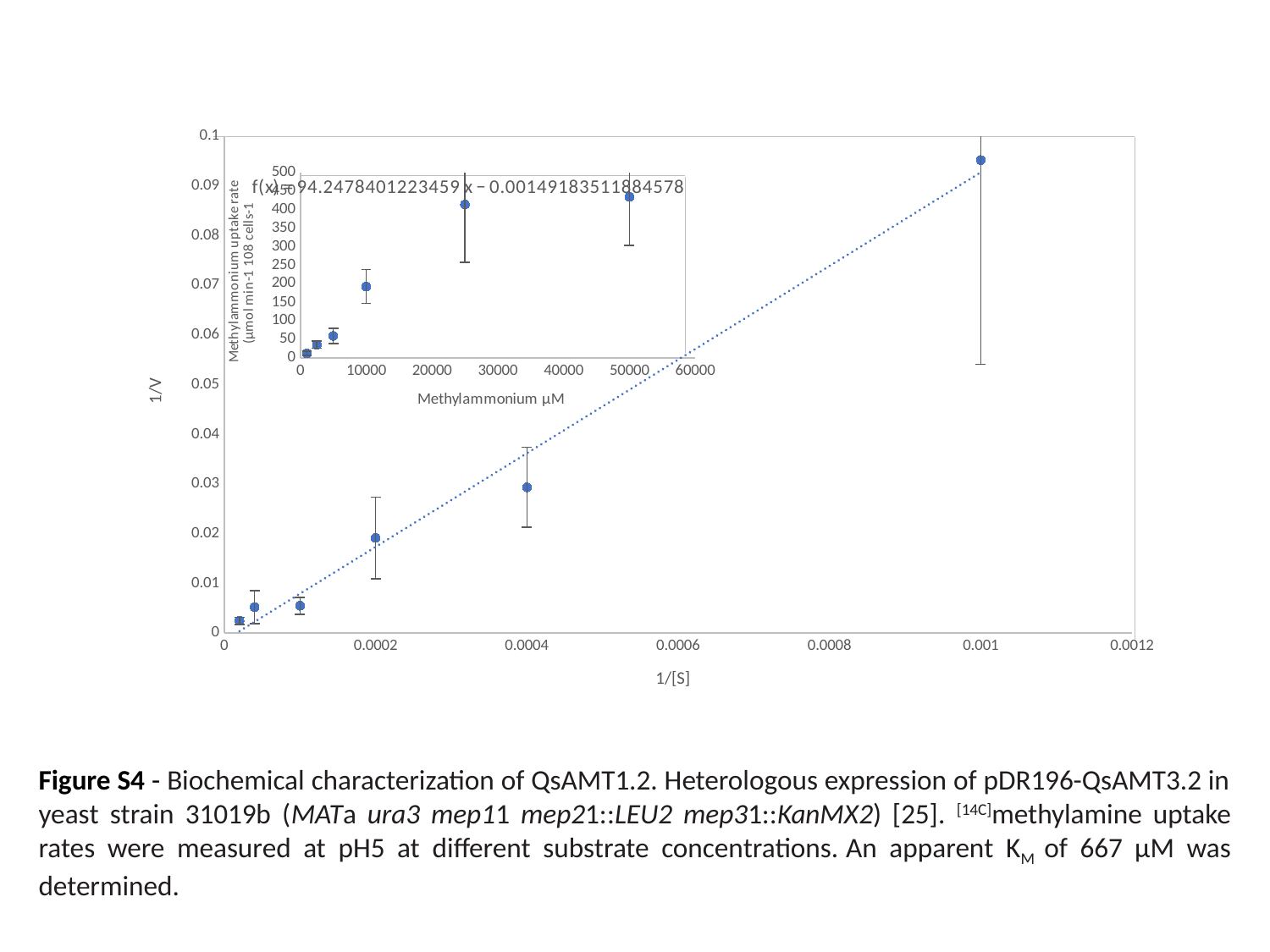

### Chart
| Category | |
|---|---|
### Chart
| Category | |
|---|---|Figure S4 - Biochemical characterization of QsAMT1.2. Heterologous expression of pDR196-QsAMT3.2 in yeast strain 31019b (MATa ura3 mep11 mep21::LEU2 mep31::KanMX2) [25]. [14C]methylamine uptake rates were measured at pH5 at different substrate concentrations. An apparent KM of 667 µM was determined.
