# Supplementary material for: Nitrogen Acquisition and Transport in the Ectomycorrhizal Symbiosis—Insights from the Interaction between an Oak Tree and Pisolithus tinctorius
Source: Plants (Basel). 2022 Dec 20;12(1):10. doi: 10.3390/plants12010010 (PMC9823632; doi:10.3390/plants12010010)
Supplement: Supplementary file 1 [file plants-12-00010-s001.zip › Figure S6_revised.pptx]

## Slide 1
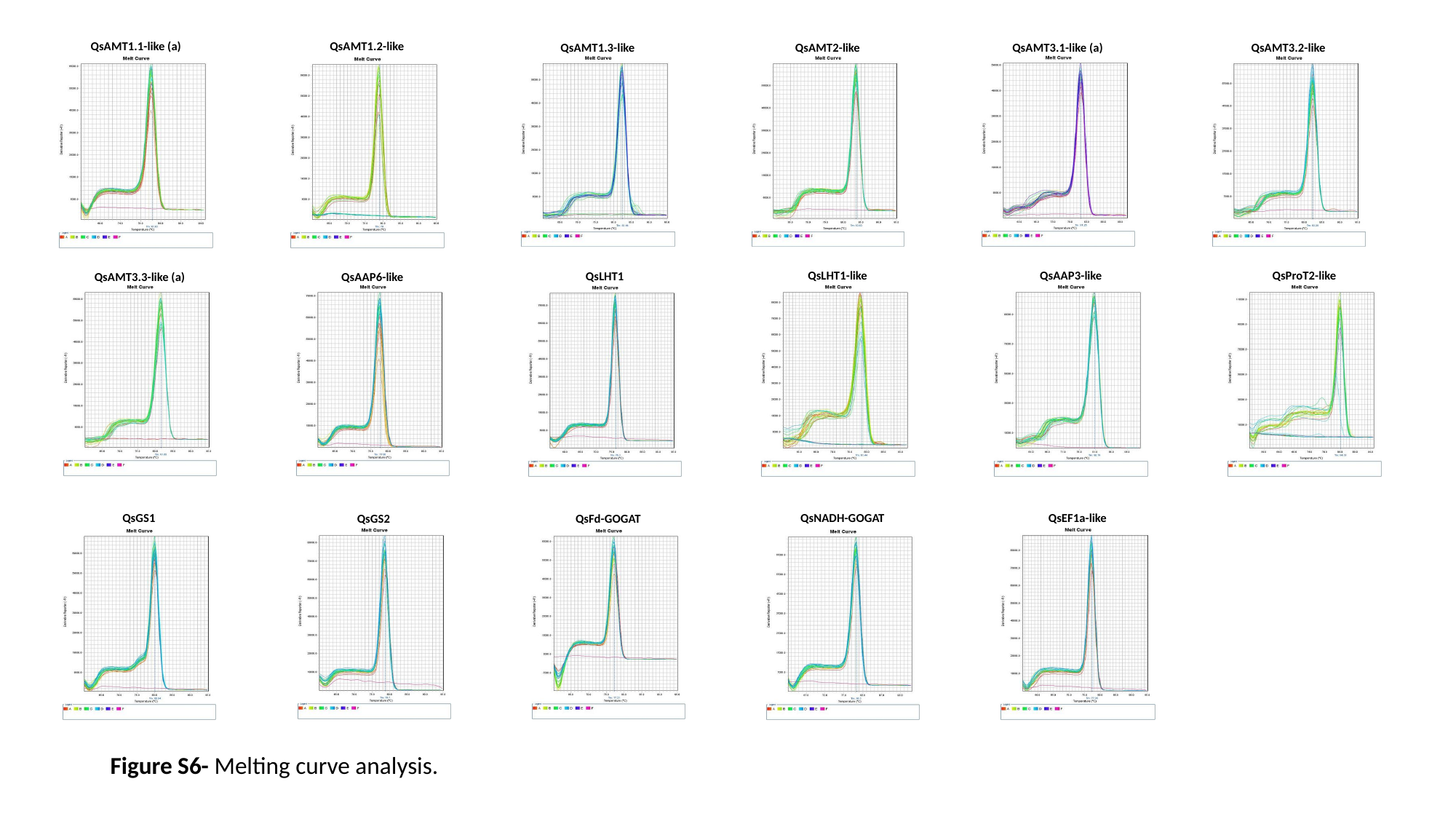

QsAMT1.1-like (a)
QsAMT1.2-like
QsAMT1.3-like
QsAMT2-like
QsAMT3.2-like
QsAMT3.1-like (a)
QsLHT1-like
QsAAP3-like
QsProT2-like
QsLHT1
QsAMT3.3-like (a)
QsAAP6-like
QsNADH-GOGAT
QsEF1a-like
QsGS1
QsFd-GOGAT
QsGS2
Figure S6- Melting curve analysis.
