# Supplementary material for: Nitrogen Acquisition and Transport in the Ectomycorrhizal Symbiosis—Insights from the Interaction between an Oak Tree and Pisolithus tinctorius
Source: Plants (Basel). 2022 Dec 20;12(1):10. doi: 10.3390/plants12010010 (PMC9823632; doi:10.3390/plants12010010)
Supplement: Supplementary file 1 [file plants-12-00010-s001.zip › Methods S1.pdf]

**Methods S1**

Labeled N uptake studies were performed using [$^{14}$C]-methylammonium (Amersham). *S. cerevisiae* uptake studies were performed with yeast cells grown to logarithmic phase. Cells were harvested at an OD600 of 0.9, washed twice in water, and resuspended in buffer A (0.6 M sorbitol, 50 mM potassium phosphate, at the desired pH) to a final OD600 of 5. Prior to the uptake measurements, the cells were supplemented with 100 mM glucose and incubated for 5 min at 30°C. To start the reaction, 100 µl of this cell suspension was added to 100 µl of the same buffer containing at least 15.8 kBq [14C]-methylammonium, specific activity 7.66 GBq/mmol (Amersham) and unlabeled amino acid to the concentrations used in the experiments. Sample aliquots of 45 µl were removed after 15, 60, 120, and 240 s, transferred to 4 ml of ice-cold buffer A, filtered on glass fiber filters, and washed twice with 4 ml of buffer A. Carbon-14 uptake was determined by liquid scintillation counting. Transport measurements were repeated independently and represent the mean of at least three experiments.

Michaelis-Menten and Lineweaver-Burk representation of the data were used to determine apparent kinetics parameters ($K_m$, $V_m$).
