# Supplementary material for: Nitrogen Acquisition and Transport in the Ectomycorrhizal Symbiosis—Insights from the Interaction between an Oak Tree and Pisolithus tinctorius
Source: Plants (Basel). 2022 Dec 20;12(1):10. doi: 10.3390/plants12010010 (PMC9823632; doi:10.3390/plants12010010)
Supplement: Supplementary file 1 [file plants-12-00010-s001.zip › Table S1.pdf]

Table S1. Root colonization, N concentration and plant growth.

	**Root colonization (%)**	**N concentration (%)**	**Height (cm)**	**Shoot FW (g)**	**Root FW (g)**
Non-Myc	0	1.75(± 0.06)	41.2(± 7.5)	20.8(± 8.1)	35.4(± 7.7)
Myc	40(± 5.8)*	1.85(± 0.05)*	51.1(± 14.9)	25.0(± 7.6)	36.8(± 9.4)

Values are the means ± standard deviations of 10 replicated plants from each treatment. * indicates significant differences between treatments at p-value <0.05.
