# Supplementary figures and images for: Nitrogen Acquisition and Transport in the Ectomycorrhizal Symbiosis—Insights from the Interaction between an Oak Tree and Pisolithus tinctorius
Source: Plants (Basel). 2022 Dec 20;12(1):10. doi: 10.3390/plants12010010 (PMC9823632; doi:10.3390/plants12010010)

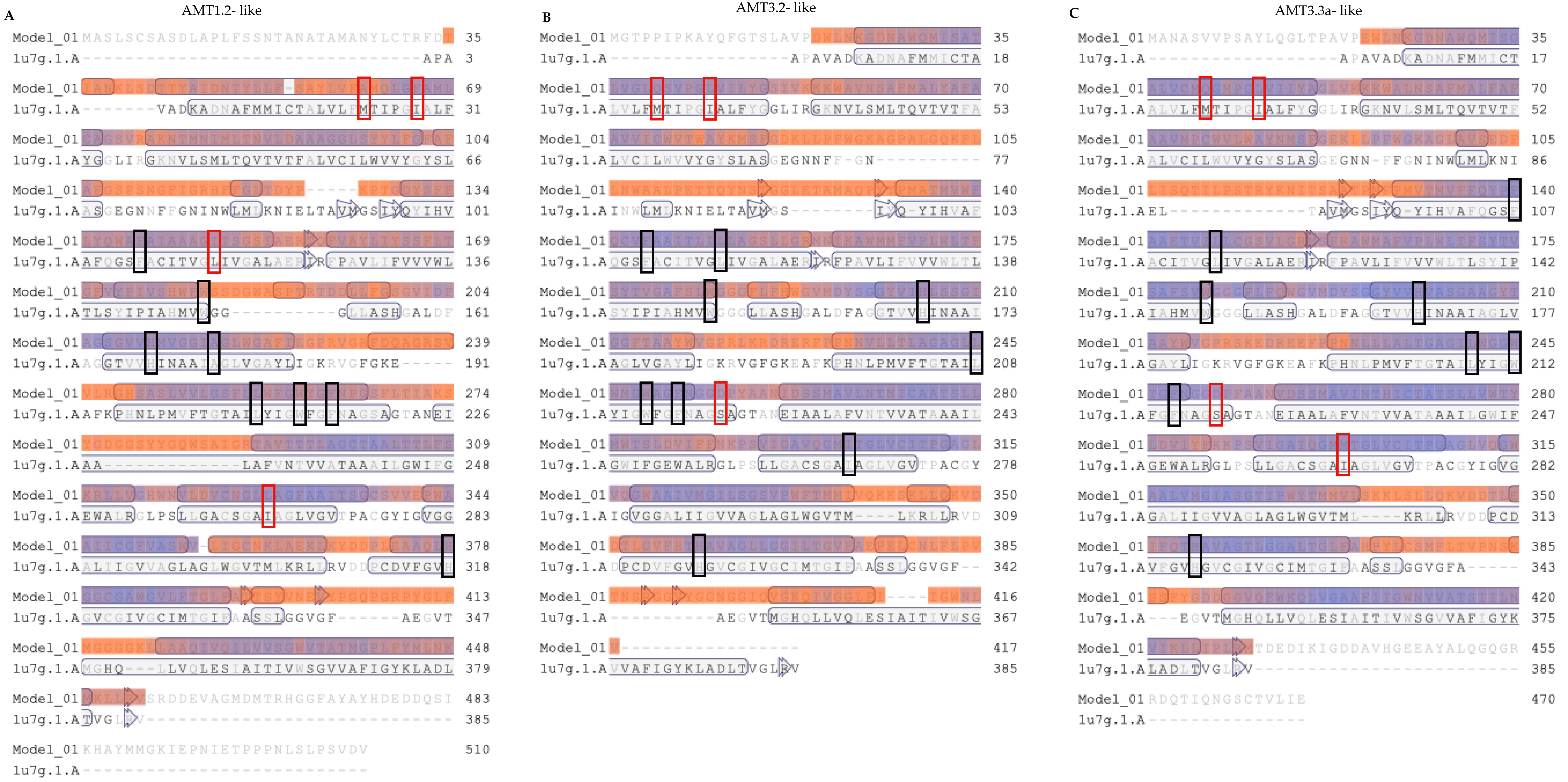

Supplement: Supplementary file 1 [file plants-12-00010-s001.zip › Figure S5.jpg]
